# Modern Sociology

**Published:** 1899-09-30

**Authors:** 


### Modern Sociology.

### THE EDUCATION OF THE MISTRESS.

Many amiable people are deeply distressed that the daughters of the humbler classes are not taught in their childhood all the details of domestic work. They talk earnestly of the necessity for educating girls for their work in life, and seem to think that it is the whole duty of school boards to turn out fully-qualified cooks and housemaids. But, whatever truth there may be in this view of the functions of the educational authorities, it is at the best but half a truth, and should be supplemented by advice as to the education of the mistress as well as of the servant.

It is doubtful if the daughters of the working man go out to service knowing less of domestic work than their mothers did; but it is perfectly certain that the daughter of the middle, at least the upper middle-class, goes into housekeeping on her marriage with much less knowledge of the management of a house and servants than *her* mother did before her. Girls stay longer at school than they did a generation or so ago, and when they leave school they are more inclined to take up some branch of study and try to excel in it than to turn their attention to the details of housekeeping. Yet they marry in due time; and, in the deepest sense, make better wives and mothers than if their whole attention in the later years of their girlhood had been given to cooking and dusting. They can sympathise with their husband's tastes and ambitions, and supervise their children's studies, as they could not have done had their previous interests been confined to the dining-room and the kitchen. But, at least when they first marry, they do not know how to manage their house; they do not know how to manage servants, and hence comes trouble.

It is true that many girls take a course of cooking lessons before they marry; but even admitting, for the sake of argument only, that "feed the brute" covers the whole duty of wife to husband, can this be done successfully merely by learning how to make pastry, and perhaps a few entrées, which will only be wanted for an occasional dinner party? Perhaps they will not turn out a success then, if the fair young cook has not previously studied her range and found out what are its peculiarities in the matter of flues and the like. "First catch your hare" applies to soup only; but "first study your range" is at the foundation of all successful cooking. It is a black business truly—black looks on the part of the mistress and maid, in addition to all possibilities of soot and dust—but the investigation of the condition of the kitchen flues is an essential preliminary to the production of dainties; and, if these be wrong, the most faithful following of the best instruction in the world will not produce good results.

And that is, after all, only one part of housekeeping. The School Board may teach cooking, and does so, with good results; but it cannot teach the work of the housemaid; nor is there in the home from which the housemaid usually comes much opportunity for learning the proper way to sweep carpets, or protect velvet-covered furniture, or dust valuable bric-à-brac. All that has to be learned in her first situation. She is fortunate if that first situation is a subordinate position in a great house where there is an experienced housekeeper to teach her her duties. But in most households it is the mistress who ought to be the teacher, and it is hard upon her maid, as well as on herself, if she is unable to give the necessary instruction. And, of course, the inexperienced mistress is at the mercy of the experienced but unconscientious servant. If she does not know how long any given piece of work takes to do, how can she tell whether the maid has been, as she says, busily employed all the time, or has been reading a penny novelette instead? How can she tell if soap and coal and other things last as long as they should? And in these things lies many possibilities of economy or extravagance. How, in short, can she be the head of a household and properly supervise the hands who do its work if she has never learned the details of management?

But how is the prospective mistress to learn. A generation ago her mother would have taught her. But now, to tell the truth, Mamma is not so domesticated in her tastes as she was, and leaves more to the servants—as she may safely do, because she knows, both from experience and from her own early training, what she ought to expect from them. And the servants, though they do not mind Miss taking care of her own room, which frees them from all responsibility regarding it, object to her hanging around looking at their work. So, in general, the poor girl is forced to wait till she has a house and servants of her own to experiment upon.

But of late years there has been attempt made to cope with the difficulties of the situation by the establishment of regular schools of domestic economy, where a lady may learn all that pertains to the working of a household, from the cleaning of the saucepans upwards. No shirking of disagreeables is allowed. It is considered to be as essential to know how to wash the aforesaid saucepans and how to clean a grate as how to decorate a dinner-table and drape a curtain. The science of the household is taught as well as its art. The pupils are at first inclined to protest against the dirty tasks, until they begin to realise how important they are. Besides, they want their "House-wife's Diploma," granted when they complete their course of study satisfactorily, and that can only be gained by going through full instruction in every part of household and laundry work. Possessed of this and the knowledge which its possession implies, a girl can cheerfully face the domestic troubles of the average housekeeper. She knows what her servants ought to do; if they do not know she can teach them; and, best of all, at a pinch she can do without them. Such a mistress is not likely to tyrannise over her servants; nor, on the other hand, is she likely to be tyrannised over by them.

Not for the bride-elect alone are these schools useful. With so many colleges for boys and girls arising all over the country, with hotels increasing on every hand, there is an increasing demand for thoroughly trained housekeepers, who can combine comfort and economy; and it has begun to be felt that such work is quite within the province of a lady. The certificate of a good training school of domestic economy is a great help to obtaining this, and many women take the course of study given there with the intention of taking up housekeeping as a profession.

